# Use of the Lean Manufacturing Principles to Improve Total Parenteral Nutrition Logistics and Clinical Outcomes in the Neonatal Patient Population

**DOI:** 10.1097/pq9.0000000000000233

**Published:** 2019-11-26

**Authors:** Christopher D. Mangum, Andrew J. Stanley, Catherine C. Peterson, Laura Biava, James Dice, Jamil Khan, Sandip A. Godambe

**Affiliations:** From the * Department of Quality and Patient Safety, Children’s Hospital of The King’s Daughters, Norfolk, Va.; † Department of Pharmacy, Children’s Hospital of The King’s Daughters, Norfolk, Va.; ‡ Department of Trauma Surgery, Children’s Hospital of The King’s Daughters, Norfolk, Va.; § Department of Neonatology, Children’s Hospital of The King’s Daughters, Norfolk, Va.; ¶ Department of Pediatrics, Eastern Virginia Medical School, Norfolk, Va.; ‖ Emergency Medicine Children’s Hospital of The King’s Daughters, Norfolk, Va.

## Abstract

Total parenteral nutrition (TPN) is one of the most frequently used pharmaceuticals administered to patients in our Neonatal Intensive Care Unit (NICU). Initially, the total interdepartmental processing time (ordering, manufacturing, and delivery between NICU and Pharmacy) averaged 15.2 hours. Inefficiencies in this process only allowed TPN to infuse 8.8 hours on average before labs were collected the next morning. Given the short administration-to-laboratory collection time, we hypothesized that laboratory samples would not adequately reflect the effect of the current TPN infusion. Furthermore, clinicians would be making decisions based on suboptimal data and ultimately nourish this patient population inadequately.

**Methods:**

The project team and the frontline staff created an efficient process for the manufacture and delivery of TPN. They removed waste in the process associated with manufacturing TPN and created capacity for change upstream (ordering process) and downstream (TPN infusion process) of the internal pharmacy process. The use of selection criteria and new standard operating procedures allowed for controlled PDSA testing of changes on a subset of patients. After we attained proven, sustainable results, we scaled the improvement efforts to the entire NICU patient population.

**Results:**

After 4 cycles of change, patients now receive TPN on average 14.2 hours before new labs are collected. The interventions over the continuum of this project yielded statistically significant results, increased infusion times to our patients by 61.4% (*P* < 0.001), improved glucose homeostasis, and decreased average length of stay.

**Conclusions:**

In conclusion, creating process capacity from incremental changes and iterative PDSA cycles has yielded sustained results.

## Abstract 2

Supplemental Digital Content is available in the text.

## INTRODUCTION

The Neonatal Intensive Care Unit (NICU) premature infant population consists of our most vulnerable patients who are often dependent upon total parenteral nutrition (TPN) as their primary source of sustenance. In preterm patients, accurate and timely replication of the exchange of nourishment between mother and fetus while in utero is imperative for the growth and development of organ systems.^1,2^ This exchange is constant and allows the mother to meet the fetus’s ever-changing physiological demands. Without the appropriate balance of the macro- and micronutrients in the prescribed nutrition, adverse effects in the patient’s developmental programming could be observed later in life.^3,4^ While in utero, optimal nutrition exchange with the maternal host through the placenta has helped produce healthier babies at birth.^5^

In contrast, at our organization, we found the most recent prescription of TPN to infuse only 8.8 hours on average before the collection of laboratory samples. This infusion time did not take into account the patient’s changing physiological needs. Therefore, the focus of this quality improvement project was to use the Toyota Production System/Lean manufacturing tools to cultivate a culture of change, reduce waste in the process, and create a standard way of managing TPN. By streamlining the manufacture of TPN, reducing the risk of errors in the TPN order process (ie, transcriptions and transpositions), and creating new standards and efficiencies in the ordering process as well as the administration process, we extended the infusion time of TPN to >12 hours and simulated this natural phenomenon (ie, nutrition exchange between maternal host and developing fetus) closely to optimize patient growth and outcomes.

## METHODS

**Fig. 1.**
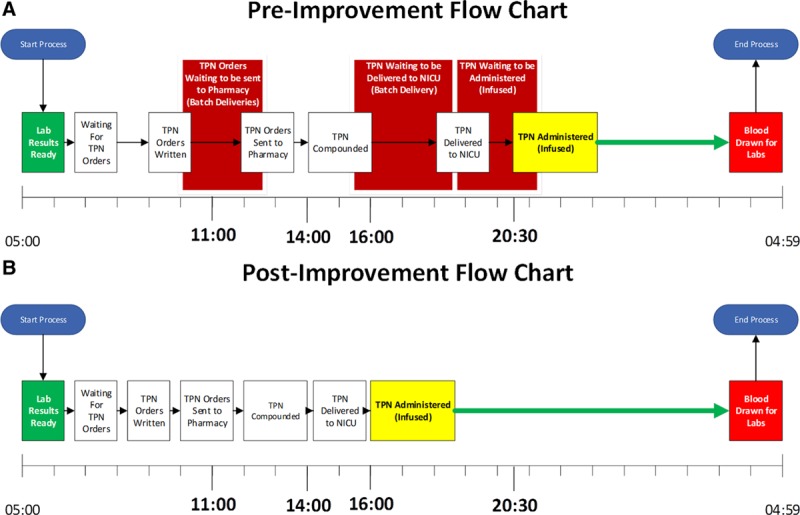
Flow chart displaying pre- and post-interventions to the throughput process of TPN. A represents the pre-improvement state. B represents the improved state (post-4 interventions).

Our team conducted this before-and-after time-series quality improvement project at a freestanding children’s hospital within a 62-bed NICU and pharmacy. The scope of this project spanned 4 phases with each depending on the previous for success. The project team completed observations to capture the baseline conditions, which included understanding the suppliers, inputs, process steps between laboratory results becoming available in the EHR and the labs (blood) being collected the next morning, the outputs of the process and finally understanding what was critical to quality (SIPOC) (Fig. 1).^6,7^ The project team also completed time studies to capture the lead time of TPN production between when staff collected morning blood samples for laboratory testing, and the time of TPN administration (Fig. 1). A value stream map was developed to display observed barriers to the continuous flow of the process and to detail the timeline of product flow between the NICU and pharmacy, respectively (Fig. 1). The value stream map was used as a guide to prioritize the areas of focused *kaizen* activity.

**Fig. 2.**
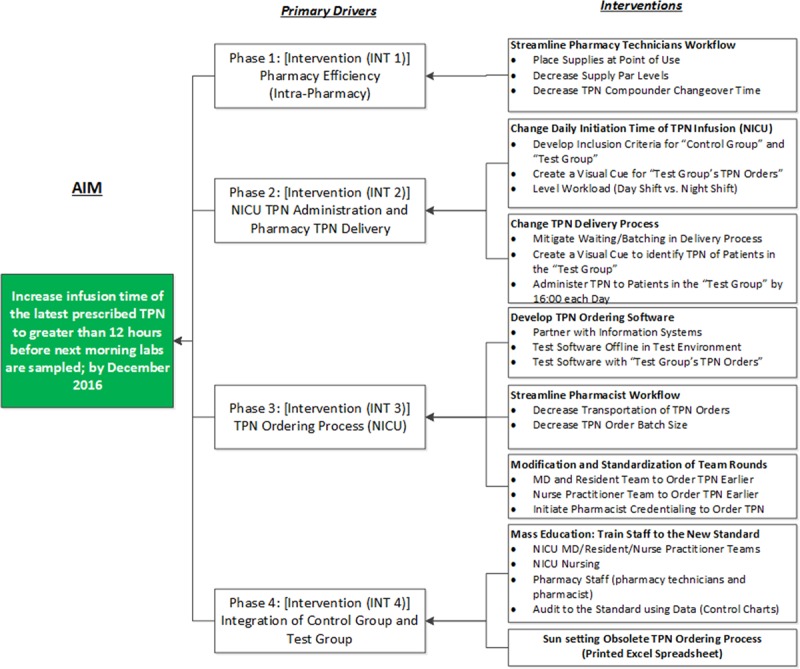
Key driver diagram detailing the scope of each phase of the project and its relevant interventions.

The project team determined that change protocols should first be implemented in the pharmacy to create capacity for change within the system. The second phase of changes targeted the TPN delivery process to the NICU and its eventual administration to the patient. These 2 initially disparate processes were aligned to reduce non-value added time. We executed interventions and created standards that detailed who should order TPN, and subsequently, how and when TPN orders were to be completed. These interventions were further assessed through small tests of change using reiterative PDSA cycles and finally implemented throughout the NICU and eventually spread to other hospital units that administered TPN. A summary of interventions is shown in the Key Driver Diagram (Fig. 2) and are explained below and later in the Results.

### Interventions

#### Phase 1: Pharmacy Efficiency (Intra-pharmacy)

The initial focus was to create capacity and capability for change within the pharmacy to assure the consistent manufacture and delivery of TPN to the NICU by 14:00 each day. This change required an examination of all work completed in the Aseptic Processing Environment for efficiency with adherence to the United States Pharmacopeia Chapter 797 guidelines.^8^ The project team observed significant variation in the daily changeover operations of the compounding machine used by pharmacy staff to manufacture TPN.

Additionally, no standard processes were noted in the stocking and restocking of components used in compounding. Therefore, our team used single minute exchange of die methodology to decrease the changeover time of the compounder.^9^ Detailed in Figure 2 “INT 1,” this methodology focused exclusively on reducing the amount of time the compounder was unavailable by streamlining what products were needed to set the compounder up and making sure supplies were available in the accurate amount, each time. The team coupled the implementation of single minute exchange of die with 5S organizational methodology. A 5S (sort, shine, set in order, standardize, sustain) tool was utilized to organize equipment and inventory in the anteroom to optimize inventory in the cleanroom and optimize personnel workflow to improve TPN supply and manufacturing logistics within the pharmacy.^7,8,10–12^ This intervention led to the creation of supply kits, which minimized storage and decreased par levels. These kits also served as a visual cue for the pharmacy technicians to know if something was missing from the kit(s) immediately. We reduced the intra-pharmacy turn-around time (IPTAT) for the manufacture of TPN and shifted our attention to the next area of focus, the NICU.

#### Phase 2: NICU TPN Administration and Pharmacy TPN Delivery

The project team’s improvement efforts shifted to the NICU’s administration process and focused on the initiation of the TPN infusions earlier in the day. As part of INT 2 (Fig. 2), Neonatologists collectively determined that the first population for the initial PDSA cycle in this phase would be defined by the following inclusion criteria: (1) the patient was beyond the first 2 days of TPN use; (2) initiation of a patient into the new process could not occur on a Saturday or Sunday; (3) the patient’s electrolyte levels needed to be stable for 48 hours; and (4) the patient was not scheduled for transfer out of the NICU within the next 72 hours. When these criteria were not met, patients were excluded from the Test Group (Initial and Expanded) of the new process testing, and remained in the Control Group, but were eventually incorporated in the fourth phase of change. Before initiation of PDSA 1 in Phase 2 of this project, the team incorporated and trained day shift nurses on several operational changes. First, the project team sought to level the workload of NICU nurses by redistributing this work to the dayshift from a task that was primarily assigned to the night shift staff. Next, within the framework of the day shift, the team instituted a new process standard of administering TPN by 16:00 each day. Lastly, nurses were required to change IV tubing every 96 hours (a secondary process change resulting from the organization’s central line bloodstream infection reduction project) to reduce the frequency of line entry.^13^ NICU personnel (N = 160) were oriented to the new standard using Job Instruction sheets. The staff were briefed on the inclusion criteria and the practice of the “blue dotting” of TPN orders [used as a visual cue to identify the Test Group’s (Initial and Expanded) TPN]. Afterward, the project team communicated the quality improvement efforts to improve the TPN delivery process to all patient families.

**Fig. 3.**
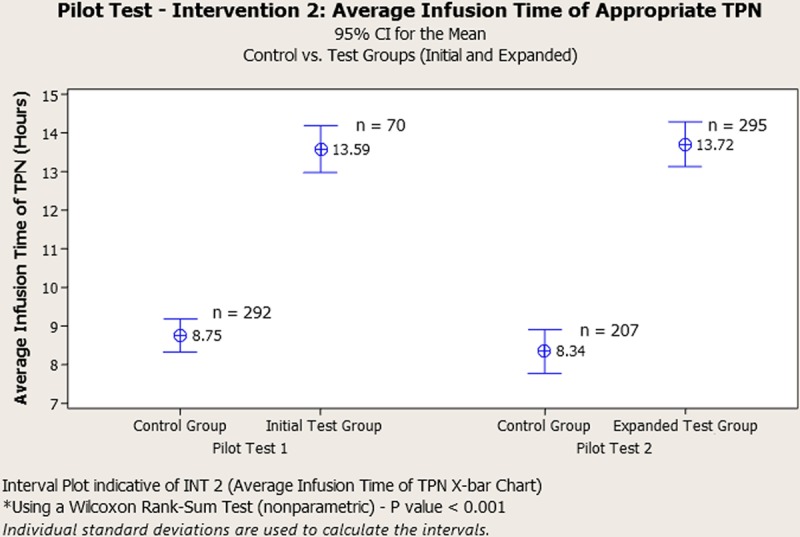
Interval plot displaying the outcomes of the first and second PDSA cycle [Intervention (INT) 2] to increase the average infusion time (AIT) of the most recent prescription of TPN. When comparing the Control Group to the Initial Test Group during the first PDSA cycle, the AIT increased by 55.3%. Similarly, when comparing the Control Group to the Expanded Test Group during the second PDSA cycle, the average infusion time increased by 64.5%, both resulting in statistical significance with a *P*-value < 0.001*.

Pharmacists processed the TPN orders identified with “blue dots” in a first-in, first-out (FIFO) fashion. After being compounded, rather than staging them for batch delivery, which occurred at 18:30 each day, they labeled the products with blue dots and delivered them as made. These changes allowed the Initial Test Group to have TPN delivered by 14:00 and administered by 16:00. The Control Group continued with the unchanged process of an 18:30 delivery time, subsequent later administration time, and shorter infusion time before the morning labs were collected (Fig. 3). The first PDSA cycle yielded the desired outcomes on a small subset of patients. To understand the impact on the interdepartmental process better, we ran a second PDSA cycle to incorporate patients meeting inclusion criteria for the entire NICU.

#### Phase 3: TPN Ordering Process (NICU)

**Fig. 4.**
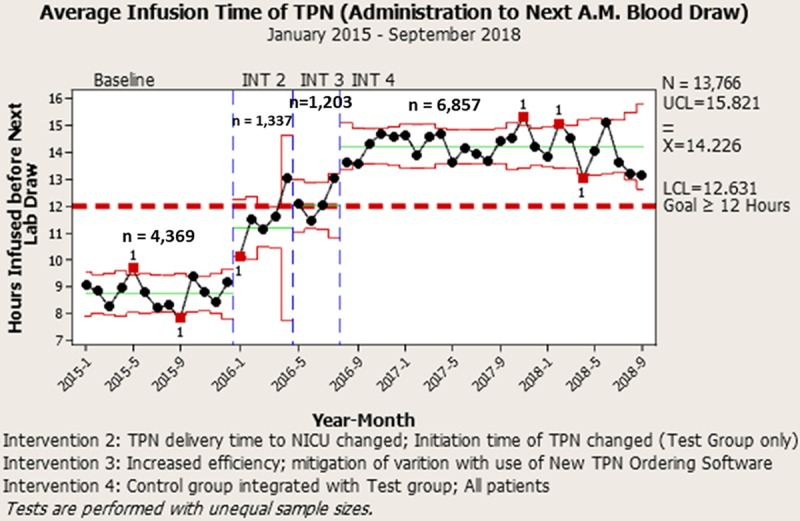
Annotated control chart (X-bar chart) displaying an increase in average infusion time (AIT) (hours) of the most recent prescription of TPN by month (sum of time in hours between the daily initiation time of TPN and the following morning’s laboratory collection time). The AIT increased by 61.4%. At baseline, the AIT measured 8.8 hours, and after 3 interventions (INT), 14.2 hours.

After the team observed TPN orders arriving in the pharmacy in batches via multiple modalities, we noted that this issue perpetuated a batched workflow throughout the rest of the internal pharmacy TPN processes, compared with the desired one-piece flow.^14^ As part of the next PDSA cycle (Fig. 4, “INT 3”), the team partnered with the organization’s Information Services (IS) department to develop and test new TPN ordering software that could be integrated into our EHR. This integration would eliminate the need for printing and completion of the archaic MS Excel TPN ordering document, and the subsequent walking to the tube station, scan station, or Pharmacy to ensure delivery of the order to pharmacy staff. Once designed, tested, and implemented, the software facilitated a consistent workflow with less manual data entry. TPN orders could now be completed easily during rounds, which created the new standard of ordering TPN before 11:00. To level the work between clinical staff (attendings, residents, and nurse practitioners) and expedite the order processing, we had the NICU pharmacists credentialed to write TPN orders through a collaborative practice agreement established with the professional staff office.

Within the Pharmacy, TPN orders arrived, and the staff processed them in the sequence received, thus mitigating batching and the varied ways in which TPN orders arrived. The newly developed software also contained improved safeguards for patient safety and reduced the amount of redundant transcription of the same TPN order throughout the original process. The approved TPN orders were sent directly to the TPN compounder for production.

#### Phase 4: Integration of the Control Group and the Expanded Test Group

With successful results from the previous iterative PDSA cycles, we combined the remaining patients from the Control Group with the Expanded Test Group (Fig. 4, “INT 4”). To accomplish this, we trained the staff assigned to the Control Group in the same fashion as the Expanded Test Group. We removed the process of identifying the Expanded Test Group TPN with the blue dot, and all NICU TPN orders were processed (ordered, compounded, delivered, and administered) the same way. To monitor the process and sustain all implemented changes, control charts (X-Bar and P-Charts) were generated and disseminated on a routine basis.

### Physiologic Findings

We proceeded to assess the effect of the TPN ordering, production, and delivery changes on the involved patients’ physiologic states by reviewing their basic metabolic panel (BMP) results. The following reference ranges of the 8 panel tests were considered when reviewing the BMP laboratory values: (1) blood urea nitrogen (3–25 mg/dL); (2) calcium (4.3–4.92 mg/dL); (3) chloride (95–105 mmol/L); (4) CO_2_ (23–33 mmol/L); (5) creatinine (≤0.6 mg/dL); (6) glucose (20–90 mg/dL); (7) potassium (4.5–7.2 mmol/L); and (8) sodium (136–145 mmol/L).^15^ Glucose homeostasis is defined as serum blood glucose levels between 50 and 125 mg/dL.^16,17^ The statistical analysis was completed using the Wilcoxon Rank-Sum Test (nonparametric) on the various BMP panels to compare pre- and post-implementation results with a Holm multiple testing correction. Additionally, the Holm multiple test correction was completed to better appreciate the statistical significance.

### Data Collection/Measures

To measure the project’s overall process and outcomes, we obtained specific data elements of the Electronic Health Record (EHR). The PowerInsight data extraction tool (Cerner Corporation, Kansas City, Mo.) was used to gather the following data elements: (1) patient demographics and characteristics; (2) admission and discharge dates; (3) hospital unit where service was provided; (4) TPN processing dates and times (order and infusion); (5) laboratory collection dates and times; and (6) serum laboratory result values for blood urea nitrogen (BUN), calcium, chloride, carbon dioxide (CO_2_), creatinine, glucose, potassium, and sodium. Additionally, the team completed manual data recording via direct observation, and self-reported logs were employed to measure process changes within the pharmacy efficiency (intra-pharmacy) stage of this project.

All data for the various interventions were collected and de-identified before analysis, which we conducted retrospectively on a monthly frequency. This quality improvement project did not meet the criteria for human subject research; therefore, review and approval by the hospital’s affiliated institutional review board was not required.

### Statistical Analysis

The project team stratified the pre- and post-intervention time study data and completed an analysis of each iterative PDSA cycle. Date and time data collected were used to calculate the following process measures: (1) intra-pharmacy turn-around time and (2) compliance with Initiating the Infusion of TPN by 16:00. The outcome measure was the average infusion time (AIT) of the most recent prescription of TPN. The balancing measures examined were the effects of the increased TPN infusion times on the basic metabolic panel tests and the Average Length of Stay (ALOS) of patients prescribed TPN in the NICU setting.

The project team also performed statistical analysis using Minitab Statistical Software v.16 (Minitab, Inc., State College, Pa.) and SAS (v 9.3) Statistical Software (SAS Institute Inc., Cary, N.C.). We considered statistical significance when *P* values were <0.05. Wilcoxon Rank-Sum Testing (nonparametric) was performed on time series data within the pharmacy to test for improved efficiency of the IPTAT and ALOS after stratification by birth weight classification. Similarly, we used the Wilcoxon Rank-Sum test to analyze PDSA tests of the average infusion time (AIT) of the most recent prescription of TPN after stratifying the data using the selection criteria. Additional analyses were performed using interval plots and control charts.

## RESULTS

**Fig. 5.**
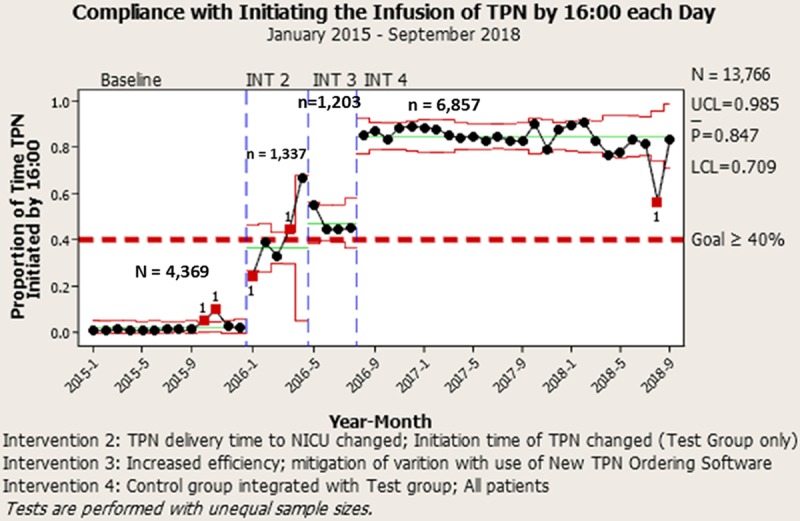
Annotated control chart (P-Chart) displaying an increase in compliance with initiating TPN infusions each day by 16:00 (sum of patients each month receiving a newly initiated TPN by 16:00 daily, divided by the total number of TPNs initiated each month). The average compliance with infusing the most recent prescription of TPN increased by nearly 2-fold. At baseline, compliance measured 2.6%, with 91.1% of TPN being administered after 20:00. After 3 interventions (INT), compliance is now 84.7%, with only 15.3% of TPN being administered after 16:00.

**Fig. 6.**
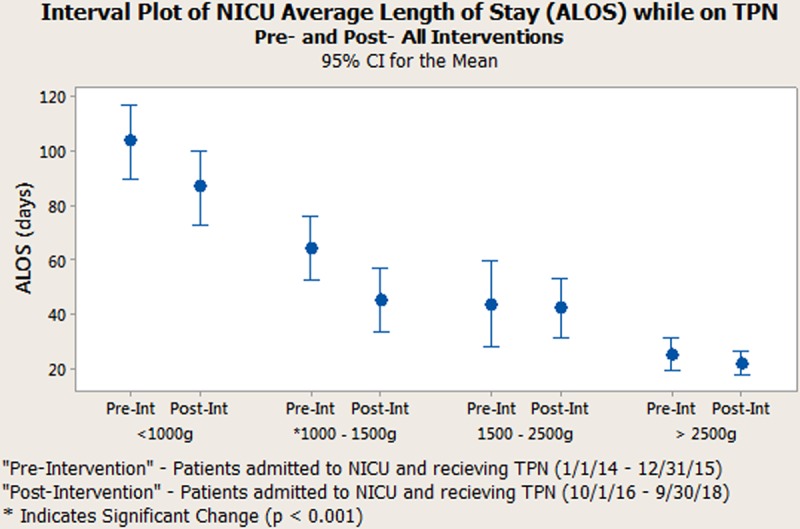
Interval Plot displaying the impact our interventions had on the NICU ALOS in patients receiving TPN. We reduced the ALOS by 20.1% and noted statistical significance (*P*-value < 0.001) between the pre- and post-intervention (pre-int and post-int, respectively) phases in patients with birthweights ranging between 1,000 and 1,500 g.

Throughout this study, 1,985 patients were admitted to our NICU, with only 722 (36.4%) receiving TPN. While working on Phase 1: [Pharmacy Efficiency (Intra-Pharmacy)] of this project, we decreased the IPTAT of TPN from 3.5 to 2.9 hours (not shown), a 17% improvement (*P* < 0.001). This improvement created the capacity to make changes in phases 2 and 3 (NICU TPN Administration and Pharmacy TPN Delivery), and [TPN Ordering Process (NICU)], respectively. In phase 2, when comparing the Control Group to the Initial Test Group, the team increased AIT during the first PDSA test by 55.31% (*P* < 0.001) (Figs. 3 and 4). During the second PDSA cycle of Phase 2, we increased the population size to understand better the impact an increased quantity of TPN orders would have on the newly designed process. Similar significance was obtained, improving the AIT by 64.5% (*P* < 0.001) when comparing the Control Group with the Expanded Test Group. The next PDSA cycle involved the TPN ordering process (NICU). We decreased the TPN transcription errors by 100%, (pre-intervention 156; post-intervention 0) (*P* < 0.001). And finally, with all changes in place, TPN now infuses 14.2 hours on average before the next morning labs are drawn (Fig. 4). Compliance and sustainability were measured throughout the continuum of the project (Fig. 5). The results of our interventions yielded improved glucose homeostasis (**see Supplemental Digital Content** at at http://links.lww.com/PQ9/A144 for Figure 1) and resulted in statistical significance (*P* < 0.001). Similarly, the ALOS decreased by 20.1% in all NICU patients receiving TPN (64.74 pre-Interventions days; 51.75 days post-Interventions) with significance found in the patient population with birth weights ranging between 1,000 and 1,500 g (*P* < 0.001) (Fig. 6).

## DISCUSSION

Safe, timely, effective, and efficient nutrition delivery to our NICU patients is crucial to improving outcomes given the catabolic burden imposed by either their premature birth, lack of adequate nutritional reserves, ongoing stressors, underdeveloped organ systems, or the presence of other comorbidities. Our improvement efforts focused on increasing TPN infusion times of the most recent prescription to replicate better the nutritional relationship present between a mother and her developing fetus. We do not fully understand the implications of non-stable levels of the elements in a basic metabolic panel but presume that glucose homeostasis is important. Episodes of hypoglycemia in preterm infants have been shown to negatively impact neuromotor and intellectual performance at 1.5 years of age.^18,19^ Alternatively, hyperglycemia in preterm infants has been associated with retinopathy of prematurity.^20^

## LIMITATIONS

The staged implementation and the analysis of physiologic data in a retrospective fashion were limiting factors of the study. The staged implementation associated with the gradual expansion of our inclusion criteria only allowed interventions on patients with more stable laboratory values and prevented us from testing the impact our PDSA cycles were making on patient physiologic data in a concurrent fashion. The initial focus of this project was to simulate the exchange of nutrition between mother and developing fetus. To accurately test this hypothesis would require routine hourly laboratory sampling to measure the intracellular ion concentrations while TPN infused. Unfortunately, this was not feasible in the inpatient hospital setting.

## CONCLUSIONS

This project embodies the values of the Toyota Production System (TPS), which is an organizational culture of highly engaged team members driving performance through the use of key technical tools to bring problems to the surface. This project reflects the value of shared learning across 2 TPS-based projects, this venture and our CLABSI-reduction project. Standardized nursing and physician practices have resulted in the use of shared Job Instruction sheets and inter-project collaboration.

Additionally, efforts to make changes in the processes associated with the ordering, manufacturing, and administration of TPN were successful. The multidisciplinary team’s collective efforts and passion led to many positive conversations about culture change from the bedside to the boardroom. This fact was evidenced not only by the sustained improvement results targeted in this project but also by the acute decrease to 0 TPN transcription errors. Furthermore, the sustained results of this TPN project were only attainable through the application of TPS methodologies, which are easily understood and mastered by frontline team members. This team and data-driven process improvement have become the new norm for the organization. Finally, we continue to assess the additional physiologic benefits of the timely infusions of TPN to vulnerable neonates. More occurrences of glucose homeostasis now occur at our organization as a result of this project, but we recommend a secondary study to understand better the complex physiology these process changes have impacted. This project demonstrates the results that can occur when frontline-driven problem solving and the expeditious surfacing of problems are encouraged.

## ACKNOWLEDGMENTS

The TPN process improvement team acknowledges the contributions of Tom Jones and Jamie Bonini, Toyota Production System Support Center, Inc. (TSSC), Plano, Tex., and Yvette M. Conyers MSN, RN, Robert J. Sepanski MS, and Allison Silva MS, BSN, RN, The Children’s Hospital of The King’s Daughters Quality and Safety Department, Norfolk, Va., for their clinical and collegial support as well as guidance past barriers on this project.

## DISCLOSURE

The authors have no financial interest to declare in relation to the content of this article.

## Supplementary Material
