# Supplementary figures and images for: Use of the Lean Manufacturing Principles to Improve Total Parenteral Nutrition Logistics and Clinical Outcomes in the Neonatal Patient Population
Source: Pediatr Qual Saf. 2019 Nov 26;4(6):e233. doi: 10.1097/pq9.0000000000000233 (PMC6946236; doi:10.1097/pq9.0000000000000233)

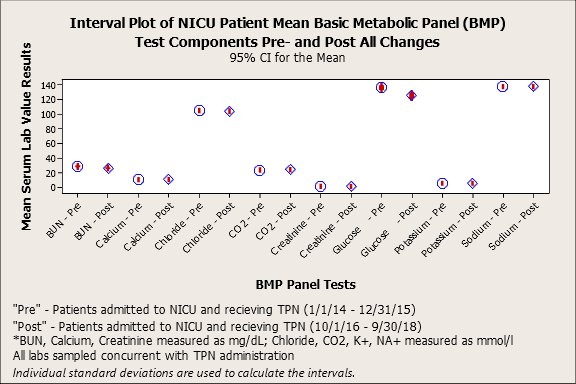

Supplement: Supplementary file 1 [file pqs-4-e233-s001.tif]

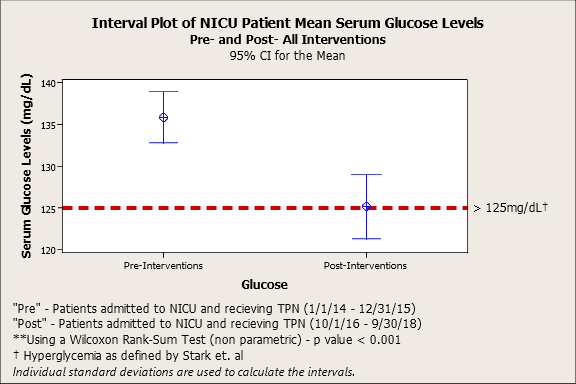

Supplement: Supplementary file 2 [file pqs-4-e233-s002.tif]
